# Nonsense-Mediated mRNA Decay, a Finely Regulated Mechanism

**DOI:** 10.3390/biomedicines10010141

**Published:** 2022-01-10

**Authors:** Fabrice Lejeune

**Affiliations:** 1 Univ. Lille, CNRS, Inserm, CHU Lille, UMR9020-U1277—CANTHER—Cancer Heterogeneity Plasticity and Resistance to Therapies, F-59000 Lille, France; fabrice.lejeune@inserm.fr; 2 Unité Tumorigenèse et Résistance aux Traitements, Institut Pasteur de Lille, F-59000 Lille, France

**Keywords:** nonsense-mediated mRNA decay, UPF proteins, regulation, gene expression, therapeutic perspectives

## Abstract

Nonsense-mediated mRNA decay (NMD) is both a mechanism for rapidly eliminating mRNAs carrying a premature termination codon and a pathway that regulates many genes. This implies that NMD must be subject to regulation in order to allow, under certain physiological conditions, the expression of genes that are normally repressed by NMD. Therapeutically, it might be interesting to express certain NMD-repressed genes or to allow the synthesis of functional truncated proteins. Developing such approaches will require a good understanding of NMD regulation. This review describes the different levels of this regulation in human cells.

## 1. Introduction

**Figure 1:**
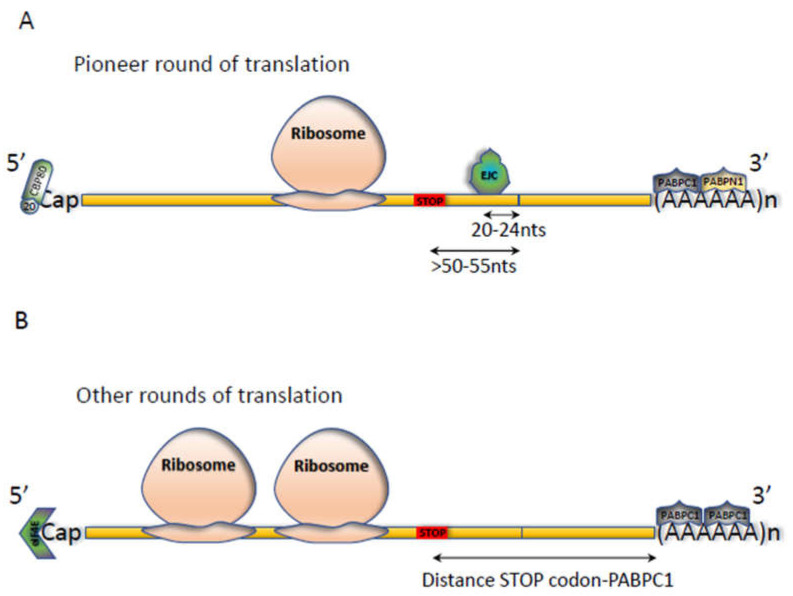
Rules for eliciting NMD according to the two models of NMD activation in human cells. **(A)** The EJC-dependent model. During the first round of translation (or the pioneer round of translation) when the CBP20 (20) -CBP80 heterodimer is present on the cap at the 5′ end and the PABPC1 and PABPN1 proteins are bound to the poly (A) tail at the 3′ end, a stop codon is recognized as a PTC if it is located more than 50 to 55 nucleotides upstream of an exon-exon junction. The presence of an EJC (exon junction complex) 20 to 24 nucleotides upstream of the exon-exon junction leads to recruitment of NMD factors when the ribosome reaches the PTC. Then NMD is activated and this mRNA is degraded; **(B)** The EJC-independent model. In this model, the distance between the first stop codon encountered by the ribosome and the PABPC1 determines whether this stop codon is premature or not.

Gene expression requires quality control at each step, from transcription to post-translational events. Nonsense-mediated mRNA decay (NMD) is certainly the most studied of all quality control mechanisms [1,2,3,4]. Its role is to detect the presence of premature termination codons (PTCs). These are identified during the first or pioneer round of translation [5] and their detection leads to activation of exo- and endonucleolytic pathways that degrade mutant mRNAs [6,7,8]. Another NMD-activating mechanism takes place during the other rounds of translation and depends on the distance between the polyA binding proteins C1 (PABPC1) and the first stop codon encountered by the ribosome (Figure 1) [9,10].

**Figure 2:**
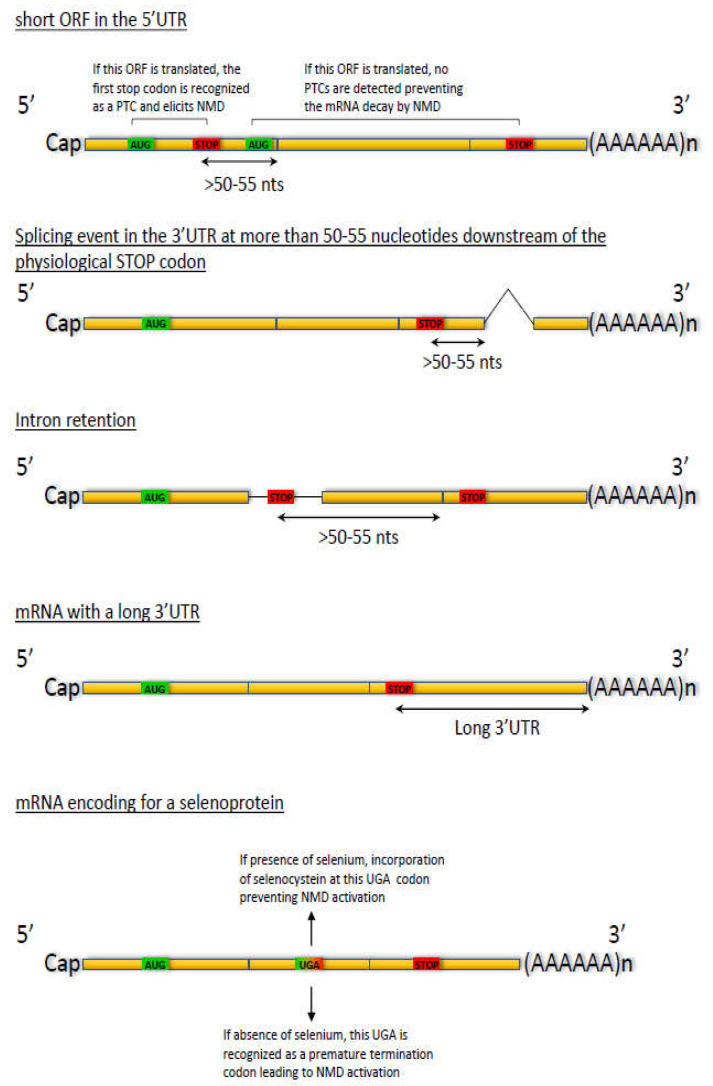
Examples of natural NMD substrates. Topmost example: Presence of a small open reading frame upstream of the main open reading frame. If the ribosome reads this small open reading frame, the stop codon of this ORF is recognized as a PTC because of the presence of downstream exon-exon junctions. Consequently, NMD of this mRNA is activated. If the ribosome reads the main open reading frame, no PTC is detected on this mRNA and its degradation through NMD is not activated. Second example: Part of the 3’UTR is removed by splicing. This splicing event results in EJC deposition 20–24 nucleotides upstream of the splicing event and hence in recognition of the physiological stop codon as a PTC if the splicing event is located more than 50–55 nucleotides downstream of the physiological stop codon. Third example: Changes in the splicing profile of a pre-mRNA lead, for example, to intron retention or exon skipping. These events very often lead to introduction of a PTC and hence to activation of NMD of this mRNA. Example four: In mRNAs having a long to very long 3′UTR, the physiological stop codon is recognized as a PTC and NMD of this mRNA is activated. Bottom-most example: mRNAs encoding selenoproteins. Selenocysteine is encoded by the UGA nucleotide triplet. In the presence of selenium and when located in a particular context allowing its recognition as the codon encoding selenocysteine, this UGA leads to synthesis of a selenoprotein. In the absence of selenium, the UGA is recognized as a stop codon and NMD of this mRNA is activated.

NMD activation thus prevents the synthesis of truncated proteins, which are very often nonfunctional or which may have acquired a function deleterious to the cell. In addition to eliminating PTC-carrying mRNAs, NMD plays a role in regulating the expression of certain genes, by modulating the level a gene’s mRNA or those of certain alternative splicing isoforms. These RNAs, called natural NMD substrates since they carry no mutation, could represent as much as 5–10% of the genes in the human genome [11], as deduced from a transcriptomic analysis following the use of a UPF1- or UPF2-mRNA-targeting siRNA. This figure might be an overestimate, however, as some transcripts whose levels are affected under these experimental conditions might not be direct targets of NMD, but rather collateral damage due to modulation of the expression of certain natural NMD substrates. There are several mechanisms through which NMD can regulate its natural substrates. Splicing events in the 3′UTR lead to deposition of the exon junction complex (EJC) downstream of the physiological stop codon, which is then recognized as a PTC. The presence of a small reading frame in the 5’UTR and changes in the splicing profile can also lead to activation of NMD of the mRNA concerned. Finally, some mRNAs with a very long 3’UTR part are regulated by NMD (Figure 2).

Because NMD is connected to diverse metabolic pathways, its activation or inhibition can have multiple consequences [12]. Very fine regulation of NMD is thus essential. As described below, NMD is regulated at different levels. This regulation can either affect the synthesis of NMD factors (from transcription to translation), and hence their stoichiometry, or it can interfere with their activity and thus affect NMD efficiency.

## 2. NMD Reaction and Regulation

### 2.1. The NMD Reaction

NMD is an mRNA surveillance process through which mRNAs carrying a premature termination codon (PTC) are rapidly detected and degraded. A PTC is a stop codon in phase with the translational start codon and located anywhere upstream of the physiological stop codon, itself in phase with the translational start codon and PTC. In mammalian cells, the position of a PTC in the open reading frame (ORF) is crucial for NMD activation. Two patterns of NMD activation presumably coexist in the cell. The first relates the position of the PTC to the presence of at least one downstream splicing event at a distance of at least 50–55 nucleotides (Figure 1A) [13]. This model involves deposition of the EJC 20–24 nucleotides upstream of exon-exon junctions as a consequence of intron splicing [14]. The EJC then recruits the NMD core factor UPF3X (also called UPF3B) and then UPF2 before UPF1 is recruited. While little is known about recruitment of UPF3X and UPF2 to the EJC, that of UPF1 has been much more studied. During the first/pioneer round of translation, the interaction of UPF1 with the CBP80 protein placed on the 5’ cap facilitates its recruitment, together with the SMG1/SMG8/SMG9 proteins and the eRF1 and eRF3 release factors [15]. This interaction then facilitates recruitment of UPF1 by the other UPF proteins (UPF2 and UPF3X) (located downstream of the PTC) to the EJC, to form the decay-inducing (DECID) complex. Interestingly, it is UPF3X which participates in recruitment of eRF3, positioning it at the A site of the ribosome [16]. UPF1 is then phosphorylated by SMG1, thus losing its affinity for SMG1/SMG8/SMG9 and the eRFs and causing their departure and recycling of the ribosome [17]. Phosphorylated UPF1, stimulated notably by its interaction with UPF2, exerts helicase activity so as to remove the proteins located downstream of the PTC, thus preparing this end for degradation by ribonucleases [18]. Phosphorylation of UPF1 increases its affinity for a protein complex composed of SMG5, SMG7, SMG6, and protein phosphatase 2A. This complex then dephosphorylates the UPF1 protein. This UPF1 phosphorylation-dephosphorylation cycle leads to activation of NMD of the mRNA, with recruitment of enzymes involved in degrading the 5 ‘and 3’ ends, such as the DCP2 decapping protein and the exoribonuclease XRN1, the deadenylation proteins PARN and CCR4, and the exosome [8,19]. In addition the SMG6 protein, thanks to its endoribonuclease activity, induces cleavage in the vicinity of the PTC, generating free, accessible 5 ’and 3′ ends for exoribonucleases [7].

The second model of NMD activation is related to the distance between the PTC and the PABPC1 protein located on the polyA tail (Figure 1B) [10,20]. The greater this distance, the greater the probability of NMD induction. In this model, the UPF proteins accumulating on the 3′UTR, particularly UPF1 [21,22,23], compete with PABPC1 located on the polyA tail for recruitment of the translation termination complex. If PABPC1 recruits this complex, termination of translation does not lead to NMD activation but leads, instead, to initiation of new translation cycles. If UPF proteins recruit the complex, NMD of this mRNA is activated and initiation of new translation cycles is blocked.

### 2.2. Tissue and Cell-Type Specificity

Upon identification of the first NMD factors in humans, expression levels of the corresponding genes were shown to differ from tissue to tissue [24]. The mRNA encoding UPF3X, for example, is abundant in the testes and fetal brain and practically absent in the fetal liver and mammary glands. These differences suggest that the NMD efficiency may vary from tissue to tissue. This has been clearly shown in the context of Schmid meta-physeal chondrodysplasia linked to a nonsense mutation in the *COL10A1* gene encoding collagen X. In patients with this disease, the mRNA encoding *collagen X* is efficiently degraded through NMD in cartilage cells but poorly or not at all in lymphoblasts and bone cells [25]. In this specific case, however, the reason for the difference in NMD efficiency from one cell type to another remains to be clarified.

Even closely related cell lines can differ in this regard. In a 2007 study, for example, up to three-fold variations in NMD efficiency were found between different HeLa cell lines from different laboratories. This variability was not due to differential levels of core NMD factors (such as UPF1, UPF2, and UPF3X), but to the EJC component RNPS1 [26].

Interestingly, correlating NMD efficiency with levels of transcripts encoding the major NMD factors appears difficult. In a study on mice, for example, nonsense-mediated decay of a PTC-carrying *MEN I* mRNA appeared twice as efficient in the ovaries and testes as in the intestines and thymus [27]. Yet, whereas levels of transcripts encoding major NMD factors and EJC components were definitely elevated in the testes, they were quite comparable in the ovaries, intestines, and thymus. This lack of correlation might mean that one or more genes whose expression correlates with NMD efficiency were excluded from this study or that NMD factor expression is regulated at different levels, as described below.

### 2.3. Regulation during Differentiation

As during cell differentiation, a new register of genes is expressed, one might expect quality control mechanisms to remain vigilant throughout this process. NMD efficiency has been studied during several cell differentiation processes, including development of the nervous system. First evidence indicates that mutations in NMD factors (including UPF3X and UPF2) or EJC proteins lead to mental retardation in humans [28,29,30,31,32,33,34,35,36,37]. Regulation of NMD during development of the nervous system seems essential, as NMD is known to regulate axonal development [38]. During this process, overexpression of the micro-RNAs miR-9, miR-124, miR-125, and miR-128 leads to repressed expression of *UPF1*, *CASC3* (also named *MLN51)*, and *SMG1* [39,40,41,42]. NMD is thus most likely inhibited, presumably to allow synthesis of natural NMD substrates.

Modulation of NMD has also been observed during differentiation of C2C12 myoblasts to myotubes. During this process the UPF2 protein level drops drastically, and this results in strong inhibition of UPF2-dependent NMD. This allows the synthesis of certain natural NMD substrates, including the mRNA encoding myogenin, a protein required for this differentiation [43].

### 2.4. Regulation through Splicing

Alternative splicing is a highly regulated process generating different mRNA isoforms from a single transcript. These different mRNA species may code for proteins with different properties. The literature abounds in reviews describing this regulation [44,45,46]. The transcripts of several NMD factors are subject to alternative splicing, including those encoding the UPF3 (also named UPF3A) and UPF3X proteins [24]. Shorter proteins may result from the absence of one or more exons (notably exons 4 and 8) in the corresponding mRNA. The specific functions of these isoforms have not yet been thoroughly investigated, although the two isoforms of have been shown to differ in their ability to interact with specific partners. For example, the short isoform of UPF3 interacts with SMG7 and little or not with UPF2, whereas the long isoform interacts strongly with UPF2 and little or not with SMG7 [47]. The fact that this exon-skipping leads to synthesis of a truncated protein rather than to a PTC-generating frameshift mutation very strongly suggests that the truncated protein must be synthesized under specific conditions and must perform a specific function that remains to be clarified.

More recently, the pre-mRNA encoding UPF1 has also been shown to undergo alternative splicing. This affects the “regulatory loop” between the UPF1 RecA2 and 1B domains, which can be either 11 or 22 amino acids long [48]. The consequence of this difference is modulation of the affinity of UPF1 for RNA and of its sequence selectivity [49].

### 2.5. Regulation Via the Endoplasmic Reticulum (ER)

There exist links between NMD and the ER. This has notably been highlighted in a recent study showing that part of the NMD machinery localizes to the translocon, which sends newly synthesized proteins to the ER lumen. NMD thus ensures quality control of the mRNAs translated there [50].

Cells undergo a wide variety of stresses, such as hypoxia, stresses induced by extracellular agents (pathogens, drugs), as well as cell-homeostasis-disrupting ER stress. If a stress is too strong or prolonged and cannot be resolved, then apoptosis is initiated. One-way cells regulate the stress response is by controlling synthesis of the factors involved in it. NMD participates importantly in this regulation, particularly in regulating levels of ER stress response factors. Very interestingly, ER stress back-regulates NMD: when ER stress becomes too great, NMD becomes unable to inhibit the stress response and this response causes NMD inhibition. This inhibition involves activation of the protein kinase RNA-like endoplasmic reticulum kinase (PERK), which phosphorylates the eIF2α protein, a subunit of the translation initiation factor eIF2 and participant in the pioneer round of translation [5,51]. Once phosphorylated, eIF2α causes inhibition of translation and hence of NMD. This might be rationalized as follows: if a stress is too strong to be coped with, the solution is to go towards cell death. This involves inhibition of NMD, leading notably to expression of apoptotic genes such as *GADD45b* and *p21* [11].

### 2.6. Regulation by Calcium

The ability of cardiac glycosides to inhibit NMD was discovered by screening the Pharmakon library, containing approximately 1,600 drug candidates. Cardiac glycosides bind to and inhibit the Na+/K+ATPase, this leading to an increase in the level of intracellular calcium [52]. Confirmation that a high cytoplasmic calcium level inhibits NMD was obtained by using an ionophore (A23187 or thapsigargin) to induce calcium release from the ER into the cytoplasm in human U2OS cells. How calcium interferes with the NMD machinery remains to be understood. One hypothesis is that an increase in cytoplasmic calcium activates caspase 3 [53], known to cleave the NMD core factors UPF1 and UPF2 during apoptosis [54,55]. It should be noted that this regulation of NMD by the intracellular calcium level might be cell type dependent: A study done on mouse N2a cells failed to demonstrate any regulation of NMD by calcium [56]. It is therefore likely that depending on how sensitive a cell type is to changes in intracellular calcium, NMD might be more or less affected. The exact mechanism that might link intracellular calcium to the NMD efficiency thus remains to be elucidated.

### 2.7. Regulation during Apoptosis

One role of quality control mechanisms in the cell is to check the conformity of an mRNA to ensure correct transmission of the message encoded in its gene. As this checking obviously costs energy, it is legitimate to wonder whether a cell engaged in a cell death program continues to devote resources to checking the integrity of mRNAs to be translated. For NMD, two teams answered this question in 2015. They found NMD to be inhibited during apoptosis, following activation of caspases 3 and 7, which cleave the two central NMD factors UPF1 and UPF2 [54,55]. Interestingly, as the fragments thus generated are themselves apoptotic, an apoptosis-amplifying loop is established. Inhibition of NMD during apoptosis thus plays both a passive and an active role: ensuring that the cell does not commit energy resources to this quality control mechanism and contributing towards the cell death objective via production of UPF1- and UPF2-derived apoptotic fragments and through increased expression of NMD-repressed apoptotic genes, such as *GADD45b* and *p21* [11].

### 2.8. Regulation in Cancer

As tumor cells divide actively, their genetic program is expressed quickly. Hence, the question: Are quality control mechanisms such as NMD optimized under these conditions, so that cancer cells are functional, or does quality control failure, for lack of time, lead to the accumulation of aberrant mRNAs liable to give cancer cells a selective advantage? There exist experimental data in favor of both of these opposing hypotheses. In hepatocellular carcinoma, decreased expression of the *UPF1* gene has been shown to result from hypermethylation of its promoter [57]. Given the central role of UPF1 in NMD, this results in decreased NMD efficiency and hence in increased levels of natural NMD substrates. One of these targets is SMAD7, which downregulates expression of *TGF-β*, predominantly a tumor suppressor gene. In this situation, NMD would thus appear to play an anti-oncogenic role.

The gene encoding the MARVELDI protein is another example, observed in lung cancer, of a gene repressed during tumorigenesis by hypermethylation of its promoter. Decreased expression of this gene impacts the interaction between SMG1 and UPF1, causing decreased NMD efficiency [58]. Again, NMD must be inhibited to allow tumorigenesis to continue.

In cancers such as microsatellite instability (MSI) cancers, NMD has been shown, on the contrary, to be activated through increased expression of certain NMD-factor genes: *UPF1*, *UPF2*, *SMG1*, *SMG6*, and *SMG7* [59]. Increased levels of these factors would appear to increase NMD efficiency. In this example, it thus appears that NMD must be made more efficient to allow cancer cells to continue tumorigenesis.

More recently, the AKT1 protein kinase has been shown to play an essential role in NMD by phosphorylating UPF1 [60]. AKT1 is known above all as a central component of the PI3K/AKT/mTOR signaling pathway, which notably stimulates cell proliferation, cell survival, and the cell cycle. Its very frequent overexpression in cancers leads to increased NMD efficiency.

Hence, depending on the type of cancer and on the expression profile of the cancer cell genome, NMD may appear as having either a tumorigenesis-amplifying or an anti-oncogenic action. This highlights the importance of NMD regulation, given the impacts of this process on cell physiology.

### 2.9. Autoregulation

It is very common for a mechanism to self-regulate. For example, the expression of transcription factors is often regulated at the transcriptional level. Likewise, many splicing factors are regulated by alternative splicing. It is therefore not surprising that several central factors of NMD are regulated by NMD. Thus, levels of the proteins UPF1, UPF2, UPF3X, as well as SMG1, SMG5, SMG6, and SMG7 rise when NMD is inhibited [59,61], so as to compensate for decreased NMD efficiency. The corresponding transcripts are natural substrates of NMD, and the underlying mechanism involves their 3’UTR, whose length exceeding 1kb puts them in the ‘long 3’UTR’ category. In addition to a long 3′UTR, the transcripts encoding UPF2, SMG1, SMG5, SMG6, and SMG7 have one or more small upstream ORFs providing another means of NMD activation [61]. The presence of several elements that make NMD-factor-encoding mRNAs natural substrates of NMD suggests a complex autoregulation which certainly must respond to specific changes in the intra- and/or extracellular environment.

## 3. Conclusions

NMD, as a quality control mechanism and as a regulatory pathway for certain genes, must be finely regulated so as not to disrupt the expression profiles of a whole set of genes and in order to respond to changes in physiological or pathological conditions. The more regulated a mechanism is, the more central its role in cell metabolism. With all the levels of regulation described above, NMD definitely falls into the category of mechanisms essential for the cell. The next challenge will be to understand how all these levels of regulation coexist. Do some regulatory pathways have priority over others? Do certain pathways respond to particular NMD-influencing parameters? What are the real impacts of NMD deregulation? Future work should aim to answer these questions. Another para-meter adds complexity to NMD regulation. NMD involves protein complexes whose protein composition varies in very specific cases [62]. It is not yet very clear whether these different complexes leading to NMD activation are all regulated in the same way or whether they allow particular sets of genes to be expressed or repressed according to the physiological conditions. This last hypothesis is supported notably by a study that measured the differential expression of NMD factors between different cell types and during development, in the absence of the UPF3X protein [61].

## 4. Prospects for Therapy Development

With all these levels of regulation, might NMD become a therapeutic target? The answer is certainly yes, because NMD can sometimes magnify the consequences of a nonsense mutation occurring at a position allowing synthesis of a partially or fully functional truncated protein. For instance, inhibiting NMD could be an interesting anticancer approach, making it possible to restore the expression of a PTC-carrying tumor suppressor gene, to express natural NMD substrates with apoptotic activity, or to induce the expression of neo-antigens on the surfaces of cancer cells [63]. NMD inhibition could also be a way to treat genetic diseases when a nonsense mutation is the cause of the disease, provided that the truncated protein synthesized upon inhibition of NMD retains partially or totally the function of the wild-type protein. It is clear, however, that while NMD is at the crossroads of many metabolic pathways [12], it is itself regulated at multiple levels. Therefore, any attempt to modulate NMD efficiency will have to be accompanied by general monitoring in order to detect impacts on metabolic pathways. This type of therapeutic approach is unlikely to fail, since one NMD inhibitor, namely caffeine, is consumed by millions of people every day and another, amlexanox, is used to treat canker sores and some forms of asthma [64,65], without any harmful effects on the body. It should be noted, however, that these are relatively weak NMD inhibitors. NMD inhibition is thus not necessarily incompatible with therapy development, but it is essential to determine the maximum level of NMD inhibition that can be achieved without risking adverse effects. It is important to identify and study new NMD inhibitors so as to establish the features that make an NMD inhibitor worthy of consideration for therapeutic development. The use of antisense oligonucleotides directed against NMD factors might thus be an effective alternative to the use of small chemical molecules, with more specific effects and a limited impact on the transcriptome [66].

It might also be interesting to consider the inhibition of NMD as a therapeutic approach for cancer to be combined with immune checkpoint inhibitors [67] or for genetic diseases to be combined with readthrough molecules. For example, PTC readthrough leads to the incorporation of an amino acid when the ribosome reaches a PTC, so that translation of the original open reading frame continues and a full-length protein is synthesized [68,69]. Readthrough thus occurs on mRNAs carrying a PTC and having escaped NMD. Combining the inhibition of NMD with the activation of PTC readthrough should make it possible to increase the quantity of RNA substrates for readthrough and thus to increase the synthesis of full-length protein molecules from the PTC-carrying mRNA.
